# Critical power: a paradigm‐shift for benchmarking exercise testing and prescription

**DOI:** 10.1113/EP091126

**Published:** 2023-01-31

**Authors:** David C. Poole, Andrew M. Jones

**Affiliations:** ^1^ Department of Kinesiology Kansas State University Manhattan KS USA; ^2^ School of Sport & Exercise University of Exeter Exeter UK

**Keywords:** athletic performance, critical power, critical speed, exercise therapy, exercise tolerance, exhaustion, oxygen uptake

1

The maximal oxygen uptake (${\dot{V}}_{O_{2}\max}$) as determined during large muscle mass exercise, e.g., running or cycling, assesses the organism's maximal capacity for O_2_ transport and utilization. Whereas ${\dot{V}}_{O_{2}\max}$ may be an excellent predictor for all‐cause mortality, even above smoking, hypertension and blood lipids (Harber et al., 2017), the tacit presumption has long been that it is the supreme arbiter of athletic performance (Saltin & Astrand, 1967). However, the triumph of elite distance runners such as Frank Shorter, Alberto Salazar and Peter Snell historically (Foster, 2019), all of whom had comparatively modest ${\dot{V}}_{O_{2}\max}$ values in the low 70s (ml O_2_/kg/min), suggests other physiological parameters may be of commanding importance for endurance exercise performance. This notion is supported by the long‐time observation that exercise training programmes produce typically small (i.e., 10–20%) and highly variable increases in ${\dot{V}}_{O_{2}\max}$ in association with far greater (i.e., 100–400%), and not always directly proportional, improvements in high intensity exercise performance (reviewed by Collins et al., 2022). One candidate parameter to explain this discrepancy is the positioning (relative to ${\dot{V}}_{O_{2}\max}$) of critical speed (CS; or critical power, CP), which demarcates heavy (i.e., sustainable) from severe intensity exercise, where blood lactate increases inexorably, ${\dot{V}}_{O_{2}}$ is driven to ${\dot{V}}_{O_{2}\max}$ and exhaustion occurs at *T*_lim_ according to Poole et al. (2016):

$$T_{\lim}=W^{\prime }/\left(P-\mathrm{CP}\right)$$

where, for cycling exercise, *P* is the extant power and *W′* denotes a non‐renewable energy store. Recently, Collins et al. (2022) have demonstrated that relating exercise training intensity to CP explains substantially more of the physiological variability and adaptations to training as well as severe intensity exercise tolerance than does ${\dot{V}}_{O_{2}\max}$.

Inherent in the work of Collins et al. (2022) is the notion that prescription of exercise intensity relative to CP (and also the gas exchange threshold, GET; see Lansley et al., 2011) will reduce the variability in physiological responses and exercise tolerance. And that is precisely the hypothesis tested by Meyler et al. (2023) in this issue of *Experimental Physiology*. Specifically, in healthy men and women who completed multiple exhausting cycling tests (graded exercise test, series of constant‐power tests) to define GET, CP and *W′*, these parameters and also ${\dot{V}}_{O_{2}\max}$ (i.e., at defined percentages of ${\dot{V}}_{O_{2}\max}$) were utilized to anchor separate moderate‐ (MOD), heavy‐ and severe‐intensity criterion exercise bouts. Subsequently, two series of MOD, HEAVY and SEVERE exercise bouts, the latter as high intensity 5 × 3 min intervals, were prescribed ‘traditionally’ (TRAD) based upon ${\dot{V}}_{O_{2}\max}$ (i.e., 50%, 77% and 85%) or using the GET and CP parameters (THR; MOD: 30 min at 90% GET; HEAVY: 20 min at 50%∆ (midway between GET and CP); and SEVERE: at 110% CP). Primary results supported the hypothesis for HEAVY and SEVERE exercise with respect to reduced variability of work rates for THR versus TRAD. Crucially, whereas some subjects for HEAVY–TRAD exercised in the severe‐intensity domain with the attendant ${\dot{V}}_{O_{2}}$ and [lactate] consequences – and 70% of them became exhausted for TRAD versus 0% for THR – this was not the case for HEAVY–THR. For the SEVERE trials neither peak nor mean ${\dot{V}}_{O_{2}}$ and heart rate variability were different between TRAD and THR, but peak and mean [lactate] variability were lower for THR, as was percentage *W′* depletion.

The scientific literature is, unfortunately, replete with examples of otherwise very well designed studies where the investigators have chosen to ‘normalize’ heavy exercise intensity by exercising their participants at either a fixed %${\dot{V}}_{O_{2}\max}$, often 75% or 80%, or at 50%∆ between GET and ${\dot{V}}_{O_{2}\max}$. So doing has resulted in highly divergent physiological responses characteristic of some participants exercising in the heavy (i.e., stable ${\dot{V}}_{O_{2}}$ and [lactate] profiles, *T*_lim_ >> 30 min) and others in the severe (i.e., ${\dot{V}}_{O_{2}\max}$ achieved, rising [lactate], exhaustion < 20 min) exercise domains. Meyler et al. (2023) demonstrate that prescribing exercise relative to CP can prevent this occurrence and, even for supra‐CP exercise, reduce inter‐subject response variability for key physiological indices. This rationale has also been presented, on a more theoretical level, for normalizing the assessment of exercise for therapeutic efficacy across patient populations, especially chronic obstructive pulmonary disease (Whipp & Ward, 2009). Although beyond the limits of the measurements in Meyler et al. (2023), it is also important to note that, by registering severe‐intensity exercise bouts to CP and thereby lowering inter‐subject variability for *W′* depletion, it would be expected that greater homogeneity would be achieved across participants for intramyocyte perturbations (e.g., ∆[PCr], [P_i_], [ADP_free_], [H^+^], [glycogen]; Jones et al., 2008) in addition to, of course, improved ability to predict exercise tolerance. The implications and therefore putative impact of the findings of Meyler et al. (2023) are substantial and reach across the preserves of exercise science to better inform, prescribe and evaluate athletic training regimens, improve the assessment of the efficacy of therapeutic interventions and, of course, help resolve the mechanistic bases for exercise intolerance itself.

## AUTHOR CONTRIBUTIONS

Both authors have read and approved the final version of this manuscript and agree to be accountable for all aspects of the work in ensuring that questions related to the accuracy or integrity of any part of the work are appropriately investigated and resolved. All persons designated as authors qualify for authorship, and all those who qualify for authorship are listed.

## CONFLICT OF INTEREST

None declared.

## FUNDING INFORMATION

None.
